# Generation and characterization of a SIVmac239 clone corrected at four suboptimal nucleotides

**DOI:** 10.1186/s12977-015-0175-3

**Published:** 2015-06-16

**Authors:** Christine M Fennessey, Carolyn Reid, Leslie Lipkey, Laura Newman, Kelli Oswald, Michael Piatak, James D Roser, Elena Chertova, Jeremy Smedley, W. Gregory Alvord, Gregory Q Del Prete, Jacob D Estes, Jeffrey D Lifson, Brandon F Keele

**Affiliations:** Retroviral Evolution Section, AIDS and Cancer Virus Program, Leidos Biomedical Research, Inc., Frederick National Laboratory for Cancer Research, Building 535, Rm. 408, Frederick, MD 21702-1201 USA; Laboratory Animal Sciences Program, Leidos Biomedical Research Inc., Frederick National Laboratory for Cancer Research, Frederick, MD USA; Washington National Primate Research Center, University of Washington, Seattle, WA USA; Statistical Consulting, Data Management Services, Leidos Biomedical Research Inc., Frederick National Laboratory for Cancer Research, Frederick, MD USA

**Keywords:** SIV, Evolution, Models of AIDS

## Abstract

**Background:**

SIVmac239 is a commonly used virus in non-human primate models of HIV transmission and pathogenesis. Previous studies identified four suboptimal nucleotides in the SIVmac239 genome, which putatively inhibit its replicative capacity. Since all four suboptimal changes revert to the optimal nucleotide consensus sequence during viral replication in vitro and in vivo, we sought to eliminate the variability of generating these mutations de novo and increase the overall consistency of viral replication by introducing the optimal nucleotides directly to the infectious molecular clone.

**Results:**

Using site directed mutagenesis of the full-length/nef-open SIVmac239 clone, we reverted all four nucleotides to the consensus/optimal base to generate SIVmac239Opt and subsequently tested its infectivity and replicative capacity in vitro and in vivo. In primary and cell line cultures, we observed that the optimized virus displayed consistent modest but not statistically significant increases in replicative kinetics compared to wild type. In vivo, SIVmac239Opt replicated to high peak titers with an average of 1.2 × 10^8^ viral RNA copies/ml at day 12 following intrarectal challenge, reaching set-point viremia of 1.2 × 10^6^ viral RNA copies/ml by day 28. Although the peak and set point viremia means were not statistically different from the original “wild type” SIVmac239, viral load variation at set point was greater for SIVmac239WT compared to SIVmac239Opt (p = 0.0015) demonstrating a greater consistency of the optimized virus. Synonymous mutations were added to the integrase gene of SIVmac239Opt to generate a molecular tag consisting of ten genetically distinguishable viral variants referred to as SIVmac239OptX (Del Prete et al., J Virol. doi:10.1128/JVI.01026-14, 2014). Replication dynamics in vitro of these optimized clones were not statistically different from the parental clones. Interestingly, the consistently observed rapid reversion of the primer binding site suboptimal nucleotide is not due to viral RT error but is changed post-integration of a mismatched base via host proofreading mechanisms.

**Conclusions:**

Overall, our results demonstrate that SIVmac239Opt is a functional alternative to parental SIVmac239 with marginally faster replication dynamics and with increased replication uniformity providing a more consistent and reproducible infection model in nonhuman primates.

**Electronic supplementary material:**

The online version of this article (doi:10.1186/s12977-015-0175-3) contains supplementary material, which is available to authorized users.

## Background

Modeling HIV disease using non-human primate (NHP) models is an essential tool to gain a better understanding of viral transmission, pathogenesis, and to evaluate treatment and prevention approaches, etc. One of the most commonly used viruses for this purpose is SIVmac239—a viral clone that yields high viral loads and causes progression to AIDS in rhesus macaques. One potential weakness in using SIVmac239 is that the standard infectious molecular clone of SIVmac239 bears four suboptimal nucleotides in its genome [1] that may increase experimental variability as the virus must first generate the corrected mutations and then these corrected viruses must be selected over time. These four, suboptimal nucleotides in SIVmac239 were identified over a decade ago [1] and are presumed to be lab-generated errors originating during the cloning process or minor variants found at the terminal stages of disease in the animal from which SIVmac239 was derived. These four suboptimal mutations are found in the primer binding site, Pol (RT and Int), and Env (overlapping with second exon of Tat and Rev) [1]. Importantly, the primer binding site mutation results in a mismatch to tRNA_3_^Lys^, potentially reducing efficient binding of the tRNA primer and thereby limiting or delaying reverse transcription [2]. It has also been postulated that tRNA_5_^Lys^ could be used as primer for lentiviral RT initiation [3], but with reduced replicative capacity. Furthermore, it is unclear if there is sufficient expression of tRNA_5_^Lys^ in host cells [4] and additional evidence indicates that tRNA_5_^Lys^ was not detected in viral genomes [5]. The suboptimal nucleotide in Env represents a synonymous mutation in gp41, but causes non-synonymous mutations in both Tat and Rev. The two Pol mutations lie in RT and Int and are presumed to interfere with proper protein folding, although the exact mechanism by which these nucleotides adversely effect viral replication is not known.

Although the suboptimal mutations can be found in a small fraction of divergent SIV lineages [3] and may represent natural polymorphisms, it is clear that each of the four variants reverts to the SIV optimal nucleotide containing consensus sequence upon replication in vitro and in vivo [1, 6]. Because this virus is consistently pathogenic in vivo, no effort was made to correct these four bases within the clone itself. Indeed, an additional mutation within the original clone that prematurely truncated nef was corrected [7] to restore nef function, but these additional four suboptimal (but functional) nucleotides remained.

The time required for these mutations to be corrected by random RT error varies from one animal to the next. We posit that the differences in the time it takes to generate these corrections in vivo and their relative fitness advantages can alter viral replication kinetics during primary infection, increasing the overall variability of viral replication between animals. These potential differences during primary infection can alter the dynamic balance between host and virus, thereby adding an extra, uncontrolled variable into each experiment. In high-dose challenge models, using the suboptimal virus is less likely to be problematic since the probability of generating these mutations at nearly the same time between animals increases with a higher effective dose. Since the viral reverse transcriptase makes 1–2 random errors per genome per RT cycle [8, 9], a high-dose challenge, in which many infectious events occur simultaneously, may provide each of the four mutations an opportunity to be corrected within the first few days, thereby limiting the variability between animals. Selection of each variant will then be determined by the relative fitness advantage/cost of each optimal/suboptimal nucleotide. The differences in replication kinetics caused by these suboptimal changes may be unimportant or too subtle to be detected in a high-dose model. However, since the demonstration that limited numbers of Transmitted/Founder (T/F) viruses initiate most HIV-1 infections [10–16], NHP research frequently utilizes a repeated or single limited-dose challenge model for mucosal infections [17–20] in an effort to more accurately model mucosal HIV-1 infection. In this approach, much lower quantities of SIV are used to challenge animals, which drastically reduces the probability that the suboptimal SIVmac239 nucleotides will all be corrected within the first few rounds of reverse transcription. In fact, given the random accumulation of mutations during acute infection with a single variant, it may require weeks before any of the suboptimal mutations are corrected by reverse transcription error. We hypothesize that the variation in the kinetics of reversion of suboptimal nucleotides in limited-dose challenge models using SIVmac239 influences viral replicative capacity and may inadvertently increase variability between animals. For studies where the timing of systemic dissemination or determining which virus/host interactions are critical to block infection, we postulate that the stochastic nature of when these mutations occur can drastically affect the time to systemic dissemination or the reproducibility of vaccine efficacy. Furthermore, this variability may be exacerbated by using in vitro infection-derived challenge stocks in which some viruses in the stock may already be corrected at the time of infection, while others remain suboptimal. Using this model, animals could be infected with either an optimal or suboptimal virus or by some combination thereof, effectively increasing the overall variability between animals. Here we report the generation and characterization of a SIVmac239 clone, designated SIVmac239Opt, with all four documented suboptimal nucleotides corrected. This virus showed a modest increase in replication over the original “wild type” SIVmac239 clone in vitro and in vivo, but overall was not significantly different than parental virus. However SIVmac239Opt was significantly less variable at set-point viremia following intrarectal infection than wild type SIVmac239. Use of this virus may be advantageous for various NHP studies that require greater consistency between animals using a limited challenge dose.

## Results

### Generating SIVmac239Opt

**Figure 1:**
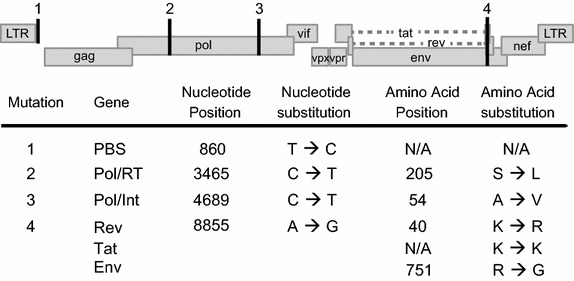
Identification of point mutations in SIVmac239WT. Schematic depiction of the SIVmac239 genome with the four mutated sites indicated. The suboptimal nucleotides are found within the 5′ LTR at the primer binding site (PBS) (*1*), in Pol (RT, INT) (*2*, *3*), and Env (Rev 2nd exon, Tat 2nd exon, and gp41) (*4*).

It has been reported that the reference clone of “wild type” SIVmac239 bears four suboptimal mutations in its genome in the 5′LTR (PBS), Pol (RT, and IN), and Env (gp41, Tat, and Rev) (Figure 1) [1, 6]. These nucleotides are rarely found in published viral sequences and revert to an SIV consensus sequence following in vitro or in vivo passage, and it has been suggested that they were artificially introduced in the process of deriving the SIVmac239 molecular clone. Here we generated a molecular clone of SIVmac239 with these sites corrected. The generation of this clone was accomplished using site directed mutagenesis with primers containing the “optimal” nucleotide designed to amplify the regions of the genome containing suboptimal nucleotides, and subsequently using Seamless technology to reassemble the fragments into one infectious molecular clone. Once the genome was reassembled, the entire viral genome was sequenced to ensure that the four suboptimal nucleotides had been changed and that no other errors had been introduced. The full genome was identical to wild type SIVmac239 (SIVmac239WT), except for the four anticipated nucleotides we modified (Figure 1). The final corrected clone was termed SIVmac239Opt.

### Protein characterization

**Figure 2:**
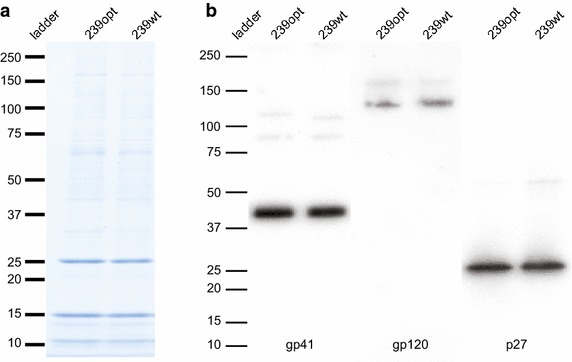
Protein analysis of SIVmac239Opt. Coomassie blue stain (**a**) and Western immunoblots (**b**) prepared from SDS-page gels of purified SIVmac239WT (239WT) or optimized SIVmac239 (239Opt). Western blots were generated by probing for gp41, gp120, and capsid p27.

Following mutagenesis, virus was produced by transfection for standard biochemical analyses of SIVmac239Opt compared to SIVmac239WT. Cell-free virus pellets were run on an SDS-PAGE gel, which was subsequently stained with Coomassie blue to compare protein expression patterns between the wild type and optimized viruses (Figure 2a). Banding patterns between wild type and optimized samples mapped identically, indicating that both the relative quantity and folding of the observed viral proteins was the same in each sample. Western blots were then performed by probing for Env (gp41, and gp120), and the p27 (CU) protein using specific monoclonal antibodies (Figure 2b). In all cases, proteins extracted from both wild type and optimized virus protein expression levels were indistinguishable.

### In vitro characterization

**Figure 3:**
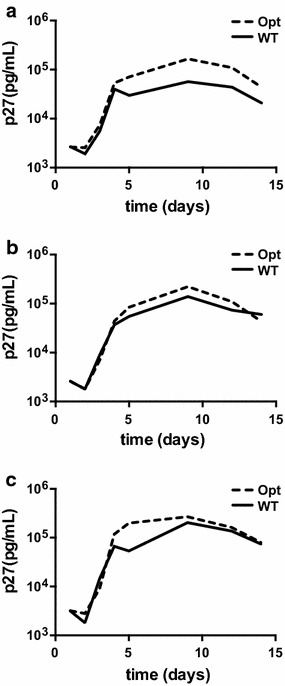
In vitro replication curves of SIVmac239Opt and SIVmac239WT. In vitro replication dynamics of SIVmac239Opt were determined by measuring p27 capsid content six times over 14 days post-infection in CD4+ T-cell enriched PBMCs from three SIV-naïve, Indian-origin rhesus macaques (**a**–**c**). Wild type SIVmac239 is displayed in a *solid line*, and the SIVmac239Opt is displayed with a *dashed line.*

To further characterize the SIVmac239Opt clone, we performed infectivity and replication assays comparing SIVmac239Opt and SIVmac239WT. The TZM-bl reporter assay was used to measure infectivity of transfection-produced stocks. Infectivity was identical between the viruses both containing 1.3 × 10^5^ IU/ml. In vitro replicative capacity was determined by infecting enriched CD4+ T cells from three independent, Indian-origin rhesus macaque PBMCs. Samples were collected every 3–4 days for 14 days, and virus measured using a SIV p27 antigen ELISA. Results from all three PBMC cultures revealed that the optimized and wild type viruses replicated at similar rates, however both peak and final set point viremia were modestly higher in samples infected with the optimized virus compared to the wild type. The peak values ranged from 8 × 10^4^ to 3 × 10^5^ in SIVmac239Opt compared with 2 × 10^4^ to 2 × 10^5^ for SIVmac239WT (Figure 3). In one PBMC culture (MacA) the SIVmac239Opt produced significantly higher p27 than wild type (p = 0.005), but this difference was not seen in the other replicates and combining all data, we found no statistical differences between viruses in vitro (p = 0.232 ANOVA).

### In vivo characterization

**Figure 4:**
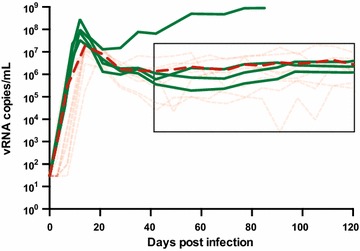
In vivo replication curves of SIVmac239Opt and wild type SIVmac239. Rhesus macaques were intrarectally infected with either SIVmac239Opt (*green*), or SIVmac239WT (*pink*). The average viral load of the macaques infected with SIVmac239WT is shown in *red*. One macaque infected with SIVmac239Opt rapidly progressed to AIDS and was euthanized at 87 days post infection.

Four purpose-bred, Indian-origin rhesus macaques were challenged intrarectally with SIVmac239Opt produced by transfection of 239T cells. The viral loads obtained by frequent blood draws were compared with those of rhesus macaques which had previously been infected intrarectally with SIVmac239 [21]. SIVmac239Opt reached peak viremia by day 12 with vRNA measurements ranging from 3.3 × 10^7^ to 2.8 × 10^8^ viral RNA copies/ml (Figure 4). Historic controls of SIVmac239 infected animals had peak viral load measurements ranging from 3.0 × 10^6^ to 7.3 × 10^7^ vRNA copies/ml. Three of the four SIVmac239Opt infected animals equilibrated to a steady state plasma viremia level of approximately 10^6^ copies/ml for over 150 days. The fourth macaque was a rapid progressor and was euthanized at day 87 following development of clinical signs of AIDS. In the historical control group rapid progressors and controllers were not included. Excluding the single rapid progressor animal in the SIVmac239Opt infected cohort, we found no statistical difference between SIVmac239Opt and SIVmac239WT in peak viral load (p = 0.086) and set point (p = 0.768). Importantly, the variance in plasma viremia levels during the viral “set point” chronic phase of infection (from day 42–150 post infection) was significantly reduced in SIVmac239Opt-infected animals compared to SIVmac239 (p = 0.005 F test to compare variance). Further, using a restricted maximum likelihood estimate of the standard deviation, we found that plasma viremia levels for wild type SIVmac239 infected animals was more than twice as variable during this phase as the SIVmac239Opt infected animals (p = 0.0015 ANOVA).

### SIVmac239OptX

**Figure 5:**
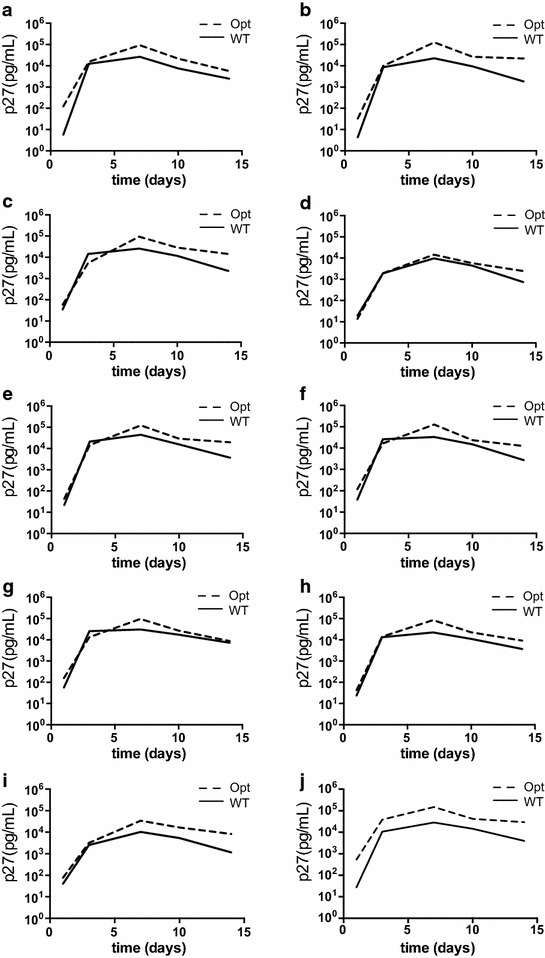
Replicative capacity of SIVmac239OptX. Replicative capacity of SIVmac239OptX was determined by measuring the p27 concentrations over 2 weeks of SupT1-CCR5 cultures infected with either individual variants of SIVmac239OptX or SIVmac239X. Optimized variants are plotted with *dashed lines* and the wild type variants are plotted with a *solid line*. **a**–**i** Depict comparisons for each pair of similarly tagged variants while **j** depicts the parental strains.

We recently reported on a pool of molecularly tagged but otherwise isogenic variants of SIVmac239 wherein 2–3 synonymous changes were introduced into the integrase gene to generate genetically distinct but biologically equivalent versions of SIVmac239 [21]. These variants were designed to be combined into a “synthetic swarm” of sequence discriminable but biologically equivalent viruses that can be used to track independent infection events in viral transmission and dissemination; detailed analysis suggested that despite the introduction of only 2–3 synonymous mutations, two of the variants showed decreased replicative capacity relative to the others (21). We transferred these same molecular tags from the wild type SIVmac239 into the optimized SIVmac239Opt clone and compared replication capacities of each tagged clone. After sequence confirming that each individual clone had only the reported mutations, each optimized clone was compared to the corresponding wild type clone for in vitro replication in SupT1-R5 cells (Figure 5). Similar to our in vitro replication curves in primary macaque cells, there were some cultures where the optimized virus significantly out performed wild type (clones A, B, H, I, and untagged with p values <0.05 for each clone), but overall, optimized clones were not statistically different from wild type clones (p = 0.377 ANOVA). Interestingly, the reduced replicative capacity of variants D and I, which were reported previously [21] was recapitulated in the optimized versions, confirming our previous report that these two genotypes, despite only three synonymous mutations, restrict viral replication.

### Primer binding site correction

**Figure 6:**
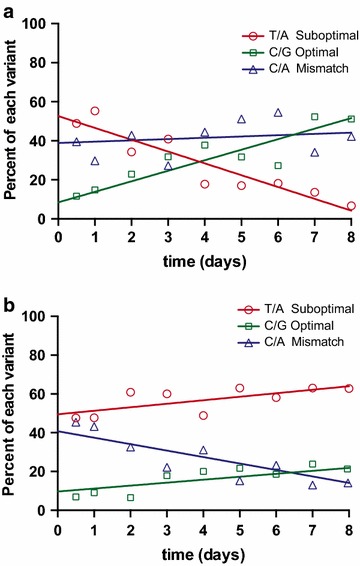
Suboptimal correction and fixation of the mutation in the primer binding site (PBS) over time. SupT1-CCR5 cells were infected with SIVmac239WT (**a**) or with a nonreplicating SIVmac239WT∆Env which was pseudotyped with SIVmac239WT Env to produce a single round infection model (**b**). Samples were collected at 12 and 24 h and then daily. The PBS of the integrated genome was sequenced by SGA. For both panels, the percent of each variant per day is plotted and a linear trend line was added for each population. The percentage of sequences bearing the suboptimal thymine/adenine (T/A) base is shown in *red*, those bearing the optimal cytosine/guanine (C/G) base is shown in *green*, and those containing the mismatched cytosine/adenine (C/A) base is shown in *blue.*

**Figure 7:**
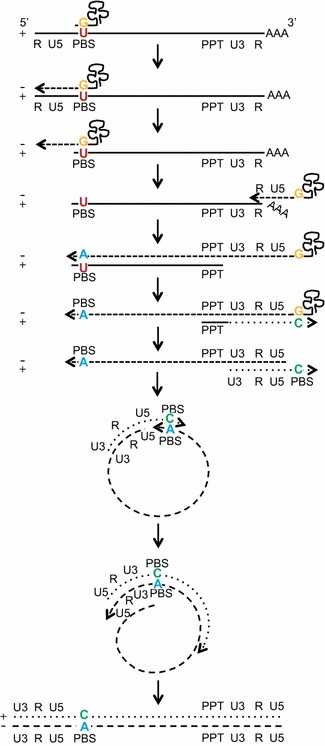
Model of tRNA-mediated mismatched integration of the PBS following viral reverse transcription. Proposed model of the method by which the mismatched pairing in the primer binding site is generated and integrated into the host genome. The tRNA_Lys_^3^ binds the viral PBS and functions as a primer for the initiation of RT but retains a mismatched base to wild type SIVmac239 with a guanine to uracil (G/U) pairing. Reverse transcription progresses with the tRNA-primed U5 region disassociating from the viral RNA PBS, and reannealing to its 3′ end and continuing transcription through the PBS. Following complete RNAseH digestion of the parental RNA (except the PPT, which subsequently acts as a reverse primer for RT), the nascent double stranded DNA circularizes and uses itself as template to complete transcription. Integration of the resulting double stranded viral DNA occurs with a mismatched base containing cytosine and adenine (C/A) at position 860 of the PBS. The base is generated from the primer is a C (*green*) and the suboptimal base is retained as an A (*blue*) on antisense strand which encodes a T in viral progeny. Length of viral genome not drawn to scale.

In order to assess the relative fitness costs of each suboptimal nucleotide, wild type virus was grown in vitro on SupT1-R5 cells for 2 months and sampled at least weekly for sequence analysis of vRNA to identify mutants and selection of optimal nucleotides. By day 21, the PBS had completely changed to the optimal version but the Env and Pol mutations had not yet arisen. While the Env mutation occurred at approximately week 10, the Pol mutations were not seen within the 2 months of this experiment. Although it is clear from published research that all four are suboptimal clones that will mutate to optimal nucleotides given sufficient time and viral replication [1, 6], the mutation with the greatest impact on early replication appears to be the PBS mutation followed by the Env mutation. In fact, the PBS mutation accumulated so rapidly that in order to precisely document this turnover we repeated the experiment sampling at 12 and 24 h and then daily for 8 days. Single genome amplification (SGA) was performed on cellular DNA to determine the proportion of sequences encoding the wild type, suboptimal thymine/adenine (T/A) pairing or the optimal cytosine/guanidine (C/G) dimer for the PBS mutation (Figure 6a). Sequence analysis from samples obtained within the first 24 h revealed that ~50% of all sequences contained the suboptimal dimer (T/A), and ~10% contained the optimal pairing (C/G). The remaining ~40% of all sequences contained a mismatched pairing of C/A at position 860 of the PBS. Since these sequences were obtained from DNA by SGA, where only a single, double stranded template is amplified for any given reaction, we concluded that following reverse transcription and integration, the viral genome contains a mismatched pairing at the suboptimal PBS position. Careful examination of the complex reverse transcription process with a tRNA_3_^Lys^ primer actually predicts a mixed base within the PBS (Figure 7). Interestingly, while the fraction of cells with mixed bases was constant over the 8-day culture, we found that the proportion of the suboptimal pairing (T/A) decreased from ~50% of the sequences down to less than 10% and the proportion of the optimal nucleotide bases (C/G) increased in the exact opposite amount from nearly 10% to over 50% (Figure 6a). The rate of loss of the suboptimal nucleotide and the reciprocal gain of the optimal nucleotide was ~5% per day.

For cells where the virus integrated as a mismatched pair, we reasoned that host repair mechanisms might edit this error by replacing either base, thereby converting the integrated viral genome into either the optimal or suboptimal form and subsequent progeny virus would contain either the optimal or suboptimal nucleotide. Alternatively, the mismatched base might not be corrected at all by the host in which case, progeny virus would be made containing the suboptimal nucleotide since the antisense strand following integration encodes for the suboptimal thymine. To elucidate the mechanism of PBS correction, we separated viral replication from host editing by infecting SupT1-R5 cells with a replication-incompetent (pseudotyped) SIVmac239. Following the single round infection, cell pellets were collected at 12 and 24 h and then daily for 8 days. Sequencing of host DNA was again performed with SGA to determine the proportion of sequences containing the suboptimal, optimal or mismatched bases (Figure 6b). As expected, the initial proportion of the group was precisely as seen in our previous experiment prior to viral replication (Figure 6a). But by day 8 without viral replication, we saw a substantial decrease in the fraction of mismatched bases from ~40 to ~10% of all sequences. Importantly, the suboptimal and the optimal nucleotides both increased at exactly the same rate (1.5% per day) over the first 8 days. Therefore, without viral replication, the host repair mechanisms correct the mismatched bases by forming either the suboptimal thymine/adenine (T/A) pairing or the optimal cytosine/guanine (C/G) pairing at the same rate.

There are two possible, but not mutually exclusive, mechanisms to explain the discrepancy in the proportion of suboptimal (50%) and optimal bases (10%) at the 12 h post infection time point found in both the infectious (Figure 6a) and the pseudotyped (Figure 6b) infection culture. This observation could be due to the presence of tRNA_5_^Lys^, a minor tRNA species initially detected in murine cells, which differs from tRNA_3_^Lys^ in five nucleotide positions, including a G to A mutation in the acceptor stem at position 69 [22]. This point mutation results in a tRNA isoacceptor stem that perfectly matches the suboptimal SIVmac239 PBS [3] allowing for the integration of a matched, but suboptimal T/A at site 860 in the PBS (Additional file 1: Figure S1A). Alternatively, a reduction in the processivity of RT that prematurely terminates the second strand synthesis prior to the mismatch can then be replaced by the PBS from the first strand allowing for the incorporation of a thymine during the final extension phase leading to a suboptimal T/A integrant (Additional file 1: Figure S1B). Furthermore, the 10% optimal G/C either originates from rapid host repair or following incomplete first strand synthesis that fails to reach the 5′ PBS and utilizes only the optimal G in the 3′ PBS (Additional file 1: Figure S1C).

**Figure 8:**
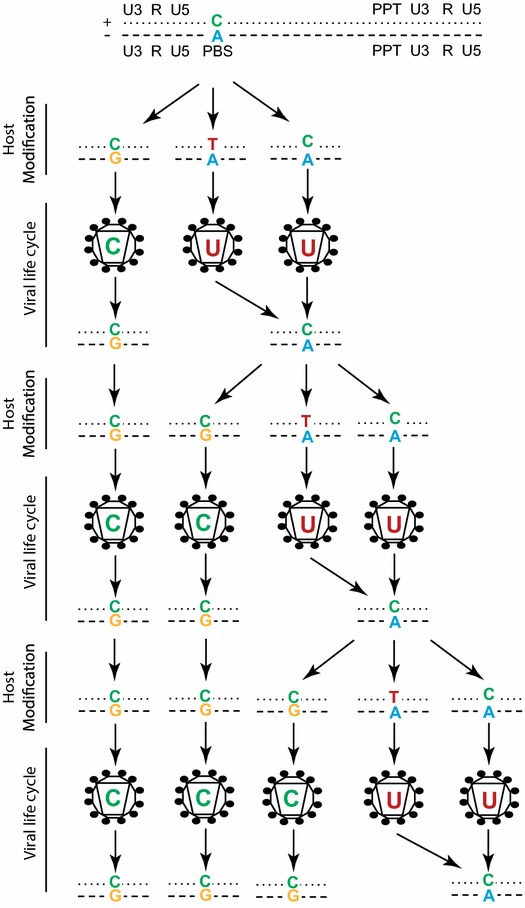
Viral genotypes resulting from transcription of integrated, mismatched viral genome. Following integration as a mismatched base, the mutation in the PBS is corrected by the host proofreading mechanism to either a matched, suboptimal thymine/adenine (T/A); a matched, optimal cytosine/guanine (C/G); or remains uncorrected as a mixed base cytosine/adenine (C/A). The resulting progeny virus produced from each of these scenarios is depicted: the matched C/G will produce virus with an optimal (C) PBS, while the mixed base (C/A), and the suboptimal (T/A) will both produce virus with a suboptimal uracil (U) at position 860 within the PBS. Virions bearing the suboptimal U in the PBS will repeat the entire cycle and integrate as matched, optimal cytosine/guanine (C/G), matched suboptimal (T/A), or mismatched as seen previously. Virions with the corrected C in the primer binding site will subsequently only integrate as a matched C/G, and will only produce progeny virus with the corrected PBS.

Regardless of the mechanism, the increased integration of the suboptimal T/A genome will produce virions with the suboptimal mutation in the PBS requiring additional rounds of replication to correct. Since progeny viruses arising from genomes retaining a mixed base will remain suboptimal, we propose a model for PBS correction with viral replication (Figure 8). For each replication cycle, progeny virus from both mismatched (C/A) and host repaired, but suboptimal (T/A) genomes will produce virus with a suboptimal PBS (uracil at site 860). Only host corrected, optimal (C/G) mutants will produce optimal progeny (cytosine at site 860) which provides the perfect nucleotide match for tRNA_3_^Lys^ binding. Each subsequent round of replication will increase the proportion of optimal mutants following host repair. During viral replication, the selection of these mutations increases due to their fitness advantage, but not the frequency in which they are generated. Additionally, given that unrepaired mismatched genomes produce suboptimal progeny, the rate of suboptimal correction is partially determined by the rapidity of host mismatch repair. Therefore, the speed by which the PBS mutant surpasses the suboptimal virus is determined by a combination the in vivo mismatch repair time of the host and the viral fitness advantage of having a matched PBS. The overall effect of the mismatched PBS/tRNA is an increase in animal to animal variation.

## Discussion

Modeling HIV-1 transmission using non-human primates is an essential tool for elucidating the all stages of the disease. High-dose infection of macaques with SIV was, until recently, a commonly used model system as it essentially ensures infection upon challenge of naïve, untreated animals [23, 24]. Recent findings suggest that a limiting-dose challenge of SIV more accurately recapitulates the transmission of HIV-1 in humans [17, 18, 25]. The experimental success of a limiting-dose challenge model is highly dependent upon a consistent infection with regular viral replication kinetics from one animal to another. Previous studies [1, 6] indicate that the most frequently utilized strain of SIVmac239 harbors four suboptimal nucleotide substitutions which may inhibit the viral replicative capacity until mutations spontaneously occur in the course of viral replication to revert these four bases to an optimal nucleotide, followed by increases in the population containing optimal nucleotides due to fitness advantages. Once fixed in the population, these mutations appear to be permanent highlighting their new, optimal state. In this study, we used basic molecular biology techniques to change the suboptimal bases in the primary SIVmac239 clone to the optimal nucleotides and subsequently characterized the modified virus in vitro and in vivo.

Our results indicated that the optimized virus showed in vitro infectivity comparable to the parental version with no obvious differences in protein production and processing. In vitro replicative capacity of SIVmac239Opt was also overall comparable to the levels observed in wild type SIVmac239. Although the optimized virus replicated better in all primary CD4+ T cell cultures, and significantly better in 1 of 3 cultures compared to the wild type virus, there were no significant differences overall. Replication in SupT1-CCR5 cells by the molecularly tagged version of SIVmac239OptX was used to compare replication of each of the ten tagged clones. We found 4 of 10 clones exhibited significant increases in replication, but these were not significant when ANOVA analysis was performed using all ten clones. In vivo experiments with the optimized virus demonstrated plasma viral loads for SIVmac239Opt were comparable to historical measurements for macaques infected with SIVmac239WT. Both peak viremia and set point viremia were not statistically different between the optimized virus and the wild type but similar to in vitro results, there were trends for higher levels in animals infected with SIVmac239Opt. Taken together, these data suggest that correcting the four suboptimal nucleotides likely increased replication capacity but with advantages not easily experimentally measured. Interestingly, the corrected nucleotides provided greater consistency during viral set point and chronic phase of infection in vivo likely due to the varying time required for changes to accumulate in different animals infected with the WT SIVmac239 containing the suboptimal nucleotides.

Our previous publication describes the benefits to inserting a silent molecular tag into the integrase gene of SIVmac239, allowing for enumeration of transmitted/founder viruses while utilizing a clonal virus [21]. In order to study transmission more accurately by removing the uncontrolled variable of suboptimal nucleotide reversion, we added the silent molecular tags to SIVmac239Opt, leaving the rest of the genome unaltered. Each individual variant was tested for infectivity and replicative competence in vitro. Each variant displayed similar kinetics to the wild type tagged virus SIVmac239X, and maybe a useful tool for studying the mechanism of transmission, dissemination, and acute replication dynamics.

Observing limited apparent replicative differences between the wild type and optimized versions of SIVmac239, we investigated how rapidly the four suboptimal nucleotides revert to their optimal base in wild type SIVmac239 infection in vitro. The primer binding site mutation originated rapidly suggesting there was a significant selective advantage of perfectly matching the tRNA_3_^Lys^ with the PBS. Integrated viral DNA was sequenced in SupT1-CCR5 cells infected with wild type SIVmac239 or a replication incompetent SIVmac239WT pseudotyped with wild type Env, with cell samples collected daily over the course of a week. We discovered that at early time points, the relative proportion of suboptimal, optimal, and mismatched bases at the PBS was identical in the sequences of the cells infected with a replication competent or pseudotyped virus. Over time in the replication competent culture, the proportion of optimal genomes increased at the same rate that the number of suboptimal genomes decreased (Figure 6a). In single round infection cultures, the proportion of optimal and suboptimal genomes increased at the same rate with the proportion of mismatched genomes decreasing (Figure 6b). We conclude from these data that rather than the expected reversion through RT error, host repair mechanisms appear to be modifying the mismatched genome, correcting it to either the suboptimal or the optimal version equally. When replication of the virus is allowed to proceed in the culture, we find an increase in the proportion of optimal genomes suggesting fitness advantage. We propose several models for explaining the mechanisms of these findings (Figure 8 and Additional file 1: Figure S1). In genomes bearing the suboptimal base in the PBS, the mismatch between the viral RNA and the host tRNA could potentially inhibit the efficiency with which the tRNA binds to the vRNA, a phenomenon which led to our initial hypothesis that the suboptimal SIVmac239 would have decreased replicative capacity. While mismatched genomes (not yet corrected by the host) and suboptimal genomes both produce suboptimal viruses, optimized genomes produce only optimized viruses and once the optimized genome is acquired, there is no mechanism to return to the suboptimal form (Figure 8). Therefore, there is an cumulative effect of the optimized genome so that over several rounds of replication all genomes will eventually become optimized, which will occur with or without any selective advantage, because there is no mechanism for back mutations to the suboptimal form. Prior to this work, it was thought that this suboptimal mutation was so inefficient that selection for the optimal nucleotide occurred within weeks. However, this is not necessarily true and we have described a rare form of viral mutation that explains both the rapid accumulation of the PBS change and the limited fitness advantage we see in vitro and in vivo accompanying this change.

At the earliest time point following infection with replication competent or incompetent virus, the relative proportion of suboptimal, optimal, and mismatched bases at the PBS was identical but different than expected. Our hypothesis that mismatches in the PBS would lead to mismatched integrated genomes predicted that all genomes would be mismatched and none would be resolved if sampled at the time of integration (Figure 7). We found that only 40% of the genomes were mismatched, while 50% were still suboptimal and 10% were already optimal (Figure 6). It was unlikely that host correction occurred that rapidly since the rate of change in the replication incompetent cells was only 1.5% per day. There are two possible mechanisms to explain the fraction of each genome which are diagramed as models in Additional file 1: Figure S1. The first model might explain the retention the suboptimal genome in half of the infected cells sampled at 12 h post exposure. There is a minor tRNA species (tRNA_5_^Lys^) originally detected in murine cells, which differs from tRNA_3_^Lys^ in 5 nucleotide positions, including a G to A mutation in the acceptor stem at position 69 allowing for a perfect match with the suboptimal SIVmaxc239 [22]. This novel tRNA was hypothesized to act as a primer in SIV and HIV variants, which bear the suboptimal thymine mutation in the primer-binding site. The minor presence of this tRNA could slow the reversion of the mutation in the PBS by mitigating the selective pressure that would otherwise be caused by the absence of a perfectly complementary primer. The second model is an alternative, but not mutually exclusive, explanation for the retained 50% suboptimal genomes which includes a premature truncation of the second strand synthesis wherein the mismatched guanine is not incorporated into the 3′ end of the genome but the suboptimal thymine is incorporated with an adenine template from the 5′ PBS during the final extension prior to integration. These models could account for the higher than predicted proportion of suboptimal genomes prior to replication or host repair. Furthermore, the reciprocal truncation could account for the 10% optimal genome seen just following integration. Here the first strand truncates at the 5′ PBS before reaching the mismatched (G/U). Again, correction occurs during the final extension of the first and second strand with the optimal cytosine of the second strand acting as the correct template prior to integration. Although we have no direct evidence for these mechanisms, they have been suggested previously or are plausible since the mismatch template will be less stable and could be displaced earlier than would occur with a perfectly matched primer.

While the overall difference between replication of the wild type and the optimized SIVmac239 was not significant, both the in vitro and in vivo data suggest a modest replicative advantage for SIVmac239Opt. Despite the limited extent of this apparent advantage, we found using two statistical measures that the SIVmac239Opt was over twice as consistent with regard to viral set-point chronic phase viremia levels compared to wild type SIVmac239. There are two limitations to this study that warrant discussion. Because this proof-of-concept study was an attempt to document the functionality of the optimized clone, the total number of SIVmac239Opt infections was limited to four animals. Furthermore, one of the four animals was a rapid progressor which might be related to the modest difference in replication of the optimized virus or might reflect non-viral host factors that predispose some animals to rapid progression. Additional infection studies should allow for the discrimination of these possible explanations. Overall, utilizing SIVmac239Opt and SIVmac239OptX might benefit NHP studies (especially preclinical vaccine evaluations and transmission studies) as it removes the uncontrollable variable of suboptimal nucleotide rates of reversion.

## Conclusions

Our results demonstrate that SIVmac239Opt is a functional alternative to parental SIVmac239, with marginally increased replication capacity. The addition of a silent, molecular tag provides a useful tool in discriminating different viral lineage and replication fitness of each SIVmac239Opt clone matched the parental clones. The PBS correction is due to host mechanisms that repair mismatched bases combined with the selective advantage of a perfectly complementary primed RT reaction. Utilizing the SIVmac239Opt and SIVmac239OptX might benefit NHP studies (especially preclinical vaccine evaluations, transmission studies, and pilot projects with limited animals) as this model eliminates the uncontrolled variable of suboptimal nucleotide reversion.

## Methods

### Site directed mutagenesis

Primers were designed to introduce the desired point mutations into the genome using site-directed mutagenesis utilizing Phusion high fidelity polymerase (Thermo Scientific). The GeneArt Seamless PLUS kit was then used to assemble the PCR fragments of the SIVmac239 genome bearing the newly modified nucleotides (Life Technologies). The resulting plasmid was transformed into Max Efficiency Stbl2 cells (Life Technologies), expanded and purified by double banded cesium chloride centrifugation. Full-length genomic sequencing was performed on the final plasmid preps to confirm point mutation generation and correct assembly of PCR fragments. The resulting sequence-confirmed plasmid was designated SIVmac239Opt.

### Virus preparation

Transfection-derived virus was prepared using Mirus Trans-IT 293 transfection reagent on Hek293T cells as described by manufacturer using the wild type or optimized SIVmac239 molecular clones. Culture medium was changed 48 h post-transfection, and cell supernatants were collected at 72 h. Supernatants were passed through a 0.45 μm filter and stored at −80°C in 1 ml aliquots. Viral infectivity was determined using TZM-bl reporter cells, which contain a Tat-inducible luciferase and β-galactosidase gene expression cassette. Infectivity was determined by assessing the number of β-galactosidase expressing cells present after infection with serial dilutions of viral stocks. After dilution correction, wells containing blue cell counts falling within a linear range were averaged and used to determine the titer of infectious units (IU) per ml in the viral stock [26].

### Protein analysis

To ensure that all proteins in the newly generated SIVmac239Opt were expressed and properly folded, protein characterization was performed. New viral preps were generated using the same SIVmac239Opt clones and transfection derived viruses prepared as described above with slight variation: the cellular medium was not changed and transfected supernatants were harvested at 48 h. The resulting 30 ml of supernatant was passed through a 0.45 μm filter, and concentrated down to form a viral pellet. Quantitative measurements of viral p27 (CA) and gp120 protein in virions for determinations of gag:env ratios and estimations of Env trimer spikes per virion were determined using a dual-color fluorescent protein gel analysis. Gels with virus samples and a dilution series of purified protein standards were stained with two fluorescent dyes (Life Technologies), Pro-Q Emerald 300 (green fluorescence) to detect glycoproteins, such as Env (envelope glycoprotein complex), and SYPRO Ruby (red fluorescence) to detect all proteins, including p27 (CA). Stained gels were analyzed for fluorescence at 520 nm with UV excitation by using a ChemiDoc MP imaging system (Bio-Rad Laboratories). The gp120 and p27 contents of each virion sample were calculated by using the TotalLab densitometry software by interpolating the integrated pixel density signals from the unknown samples onto a standard curve derived from a linear regression of density values for serial dilutions of highly purified, quantitative amino acid analysis quantified standards, either recombinant vaccinia-produced HIV-1_MN_ gp120^SU^ (generously provided by B. Puffer and R. Doms, University of Pennsylvania, Philadelphia, PA, USA) or SIVmac239 cultured in human SupT1-CCR5 T lymphoblastoid cells virion-derived p27 (CA) and well-characterized reference preparations of infection-derived HIV_BAL_, HIV_NL4-3_, and SIVmac239 (provided by J. Bess and the Biological Products Core, AIDS and Cancer Virus Program, Frederick National Laboratory, Frederick, MD, USA) were included in the analysis as controls.

### In vitro replication

Replication curves were prepared by culturing CD8-depleted Indian-origin rhesus macaque peripheral blood mononuclear cells (PBMCs) in RPMI supplemented with 10% fetal bovine serum (FBS), 2 mM l-glutamine, and 100 U/ml penicillin and 100 μg/ml streptomycin (RPMI-complete), stimulated for 3 days with 5 μg/ml phytohemagglutinin (PHA) and IL-2 (100 U/ml). Stimulated PBMCs and SupT1-CCR5 cells were infected with SIVmac239WT or SIVmac239Opt at an MOI of 0.01 or 0.001 (as determined by TZM-bl). 24 h post inoculation, cell cultures were washed with phosphate buffered saline (PBS) twice and once with RPMI-complete to remove excess virus. Viral replication was monitored over 14 days by detection of the SIV p27 antigen in an enzyme-linked immunosorbent assay (ABL) according to the manufacturer’s provided protocol.

### Animals and in vivo viral load monitoring

Four purpose-bred Indian-origin rhesus macaques (*Macaca mulatta*) were housed and cared for in accordance with American Association for Accreditation of Laboratory Animal Care (AAALAC) guidelines in an AAALAC-accredited facility, and all animal procedures were performed according to protocols approved by the Institutional Animal Care and Use Committee of the National Cancer Institute under the standards of the NIH Guide for the Care and Use of Laboratory Animals. All animals were free of cercopithecine herpesvirus 1, D-type simian retrovirus, simian T-lymphotropic virus type 1, and simian immunodeficiency virus (SIV) at study initiation. Animals were genotyped for common MHC Class 1 alleles such as *Mamu*-A*01/-A*02/-B*08/-B*17 using sequence-specific priming PCR performed as previously described [27]. *Mamu*-A*01, -B*08 and -B*17 animals were excluded from this study. Intrarectal infections were performed by placing animals at ~20° down angle in an inverted Trendelenburg position (e.g. the animal’s pelvis was elevated above its head with its sternum against the table) and the atraumatic challenge was performed using 1 cc slip tip syringes (BD Biosciences) with a small amount of non-bacteriostatic, single-use, sterile lubricant. Using transfection-derived SIVmac239Opt, four macaques were challenged intrarectally (i.r.) with 9 × 10^5^ IU in a 3 ml volume. Blood draws were obtained every 2 weeks, and plasma viral loads assessed over 14 weeks by quantitative real-time PCR as previously described [28]. Historic control animals were from animals infected intrarectally with 100 or 300 IU of infection-derived virus. The animals included in this study are 24760, 26795, 26993, 27036, 27127, 27519, 27522, 27525, 27920 described in Del Prete et al. [21].

### Molecularly tagging SIVmac239Opt

Our recent publication describes the addition of a molecular tag of two or three synonymous changes to the integrase gene of SIVmac239wt resulting in ten genetically distinct but phenotypically similar clones referred to as SIVmac239X [21]. These exact integrase molecular tags were inserted into the SIVmac239Opt clone using basic restriction digestion and ligation to generate nine tagged variants of SIVmac239Opt, designated SIVmac239OptA—SIVmac239OptI. These clones were sequenced through the entire genome to ensure correct insertion of tags. Virus was prepared from all ten of the SIVmac239OptX (SIVmac239OptA—SIVmac239OptI plus SIVmac239Opt) clones using the Mirus Trans-It transfection protocol described above, and infectivity measured using the TZM-bl assay. In vitro replication curves were prepared as described above. The resulting cell-free supernatants were tested for viral antigen using the SIV p27 ELISA assay (ABL) using the manufactures’ protocol.

### Defining the in vitro reversion of four suboptimal nucleotides

SupT1-CCR5 cells were infected with transfection-produced SIVmac239WT or a replication defective pseudotyped SIVmac239 with a luciferase reporter. Virus stocks were pre-treated with DNAseI for 1 h prior to infection to eliminate contaminating plasmid DNA. Cells were inoculated at a MOI of 0.01, and were spinoculated by centrifugation for 2 h at 1,000×*g* at 25°C, and then incubated at 37°C with 5% CO_2_ for the duration of the experiment. Infected cells were collected at 12, 24 h, and every subsequent day for 8 days. Collected cells were washed 3× in PBS and DNA extracted using QIAamp DNA Mini kit (Qiagen). Temperatures during the extraction were maintained at room temperature or lower prevent DNA melting. SGA sequencing was performed by diluting template DNA such that the majority of wells contain no template and the wells with template most likely contain only a single copy [21]. Briefly, PCR was performed with 1× PCR buffer, 2 mM MgCl_2_, 0.2 mM of each deoxynucleoside triphosphate, 0.2 μM of each primer, and 0.025 U/μl Platinum Taq polymerase (Life Technologies) in a 10-μl reaction. To sequence the molecular tag contained within integrase gene, real-time PCR was performed with sense primer SIVmacIntF1 5′-GAA GGG GAG GAA TAG GGG ATA TG-3′ and antisense primer SIVmacIntR3 5′-CAC CTC TCT AGC CTC TCC GGT ATC C-3′ under the following conditions: 1 cycle of 94°C for 2 min, 40 cycles at 94°C for 15 s, 55°C for 30 s, 60°C for 1.5 min, and 72°C for 30 s. Template positive reactions were determined using a gene specific probe SIVIntP 5′-TCC CTA CCT TTA AGA TGA CTG CTC CTT CCC CT-3′ with FAM6 and ZEN/Iowa Black Hole Quencher (Integrated DNA Technologies) and directly sequenced with SIVmacIntR3 using Sanger sequencing (Life Technologies). Additional SGA sequencing was performed spanning each of the four suboptimal nucleotides to monitor reversion over time.

### Statistical analysis

For comparisons of in vitro growth kinetics, paired t-tests were used with log10 transformed p27 measurements for each macaque. An ANOVA was then performed to identify difference between macaques. The same paired t-test and ANOVA was performed for each molecularly tagged variant in SupT1-R5 cells. The mean in vivo peak (day 12) and set point (days >42) viral loads between SIV239Opt and SIV239WT were compared using a two sample t-test and the variance around the mean was determined using a F test. Furthermore, the variance during set point was determined using a restricted maximum likelihood (REML) estimate in a random effects mixed model with an ANOVA analysis was then performed testing the differences between the two mixed effects models.

## Additional files

Additional file 1:
** Figure S1.** Possible models for the various proportions of the suboptimal, optimal and mismatched integration within the PBS. Following initial integration, the proportion of suboptimal thymine/adenine (T/A) integrated in the genome is 50% whereas the optimal cytosine/guanine (C/G) is found at only 10% and the uncorrected mixed base of cytosine/adenine (C/A) is found at 40%. There are two possible models that explain the increases proportion of the suboptimal T/A integration; (A) an alternative tRNA priming where a minor tRNA_Lys_^5^ molecule initiates the RT event producing a T/A genome, and/or (B) a reduction in the processivity of RT that prematurely terminates the second strand synthesis prior to the mismatch in the PBS which then requires utilizing the 5′ PBS from the first strand allowing for the incorporation of a suboptimal T/A integrant. To account for the 10% optimal C/G genome, we propose a mechanism whereby an early truncation of first strand synthesis prior to the mismatch will utilize the second strand cytosine for the final template producing an optimal C/G form (panel C).
